# Supplementary material for: Meta-analysis of efficacy and safety of sustained release oxycodone hydrochloride rectal administration for moderate to severe pain
Source: PLoS One. 2022 Jun 27;17(6):e0266754. doi: 10.1371/journal.pone.0266754 (PMC9236234; doi:10.1371/journal.pone.0266754)
Supplement: Attachment — Submitted filename: Response to Reviewers.docx [file pone.0266754.s002.docx]

Dear Editor,

We would like to thank PLOS ONE for giving us the opportunity to revise our manuscript.

We thank the reviewers for their careful read and thoughtful comments on previous draft. We have carefully taken their comments into consideration in preparing our revision, which has resulted in a paper that is clearer, more compelling, and broader. The ollowing summarizes how we responded to reviewer comments.Below is our response to their comments. Thanks for all the help. I would like to re-submit this revised manuscript to Plos One, and hope it is acceptable for publication in the journal.Looking forward to hearing from you soon.

Best wishes,

**Reviewer #1: The article is worthwhile as it addresses important clinical issues of pain management. In my professional opinion this paper will benefit the current clinical practice, however, I feel significant issues need to be seriously addressed. One of my main concern is the definition of moderate to severe pain that is not included. As this is part of the title, author must clearly define the term as per defined in all selected papers included in the review.**

**We would like to thank you very much for your recognition of our work and valuable comments.** **We've added definitions for mild, moderate, and severe pain to the Introduction.**

**Initial paragraph in the discussion is repetitive to the information and phrases included in the introduction. Please revise the paragraph and assure that the there is no overlapping between these sections. The utilisation of short form for 1h and 3h that represent 'hour' should be avoided** **and written in full.**

**We have truncated the repetition of the description in the introduction, modified '1h and 3h' and have written in full.**

**The paper contains quite significant grammatical errors and sentences are not written in proper English, therefore, it is suggestible for the author to** **send the paper to be edited by professional native English editor. This is imperative as the paper contains information that is useful for reference in clinical practice.**

**Eg; 'On the one hand, it can avoid the influence of nausea and vomiting caused by the primary disease on drug absorption; on the other hand, after rectal administration, the drug does not pass through the liver, thereby avoiding the first pass effect of the liver and increasing the blood drug concentration; the drug does not pass through the stomach And the small intestine, avoid the influence and destruction of acid, alkali and digestive enzymes on the medicine, reduce the irritation of the medicine to the stomach and intestines, and greatly improve the bioavailability of the medicine.'**

**The information that is not properly written in professional English may impede future reference and citation, hence, affecting the quality of this paper. I believe if the paper can be improved to assure that it is publishable.**

**We have already send the paper to be edited by professional native English editor. The article has corrected grammar and other errors in the original text.**

**Reviewer #2: The authors has produce an excellent data for this study. It is a very good alternative way to administered the mentioned medicine especially for cancer patients with difficulty to swallow. This method also will reduce several unwanted effects.**

**The authors has thoroughly stated the methods. There are several minor formatting issues that need to be reviewed before being accepted.**

**We thank you for your careful review of the manuscript and your constructive comments. We have modified the format of the article as required by the journal.**
